# Standardized Approach to Robotic-Assisted Esophagectomy

**DOI:** 10.1016/j.atssr.2024.12.010

**Published:** 2025-01-24

**Authors:** Claire Perez, Lucas Weiser, Allen Razavi, Drew Bolster, Charles Fuller, Kellie Knabe, Sevannah Soukiasian, Raffaele Rocco, Andrew R. Brownlee, Harmik J. Soukiasian

**Affiliations:** 1 Division of Thoracic Surgery, Department of Surgery, Cedars-Sinai Medical Center, Los Angeles, California

## Abstract

Esophagectomy is the standard treatment for localized esophageal cancer. However, this procedure is associated with significant morbidity and mortality. There has been a growing shift toward minimally invasive techniques, which have been shown to reduce perioperative complications, shorten hospital stays, and enhance patient satisfaction while adhering to oncologic principles. In this article, we outline our standardized method for performing robotic-assisted Ivor Lewis esophagectomy.

Esophageal resection for malignant disease, first described more than 100 years ago,^1^ has evolved to improve cancer survival while reducing morbidity and mortality. Minimally invasive esophagectomy reduces perioperative mortality and improves recovery and quality of life compared with the traditional open approach.^2^ There is no consensus on the ideal esophagogastric anastomosis.^3^^,^^4^ Each of the described methods (hand-sewn, circular stapled, linear stapled, and modified Collard) have benefits and weaknesses. There are, however, agreed-on tenets of a good anastomosis to minimize postoperative anastomotic leaks, dysphagia, and strictures.^5^ This article outlines our surgical approach to a robotic Ivor-Lewis esophagectomy.

The patient was a 67-year-old man with a history of hypertension and hyperlipidemia who experienced dysphagia and food regurgitation for months. An esophagogastroduodenoscopy (EGD) revealed a long segment of Barrett esophagus along with a mass obstructing the distal esophagus. A biopsy confirmed the diagnosis of esophageal adenocarcinoma. The patient underwent neoadjuvant chemoradiation and was subsequently scheduled for an Ivor-Lewis esophagectomy. The final pathologic stage was pT3 N0.

## Technique

At our institution (Cedars-Sinai Medical Center, Los Angeles, CA), we conduct esophagectomy as a 2-stage procedure (Video). First, we perform an EGD and diagnostic laparoscopy with vascular conditioning. Metastatic disease is ruled out, and surgical resectability is confirmed. The patient is taken back to the operating room on day 2 for completion of the robotic Ivor-Lewis esophagectomy. Jejunostomy tube placement is determined on a case-by-case basis.

### Stage 1: Diagnostic Laparoscopy and Vascular Conditioning

The patient is brought to the operating room, and general anesthesia is induced. An EGD is performed to confirm the tumor’s location and assess disease progression. The patient is positioned supine, with a roll placed at the level of the xiphisternum to bring the hiatus into the operative field. A Veress needle is inserted at Palmer’s point for abdominal insufflation. Four robotic ports are marked at the costal margin: the camera port is placed approximately 1 cm to the left of the midline, followed by 2 additional 8 mm ports on the left and a 12 mm port to the right, designated for stapling during vascular transection and conduit creation. A 12 mm robotic assistant port is triangulated between the camera and the stapling ports (Figure). The left lobe of the liver is elevated using a Nathanson retractor to expose the diaphragmatic hiatus. The patient is then placed in the reverse Trendelenburg position, and the robot is docked.

**Figure:**
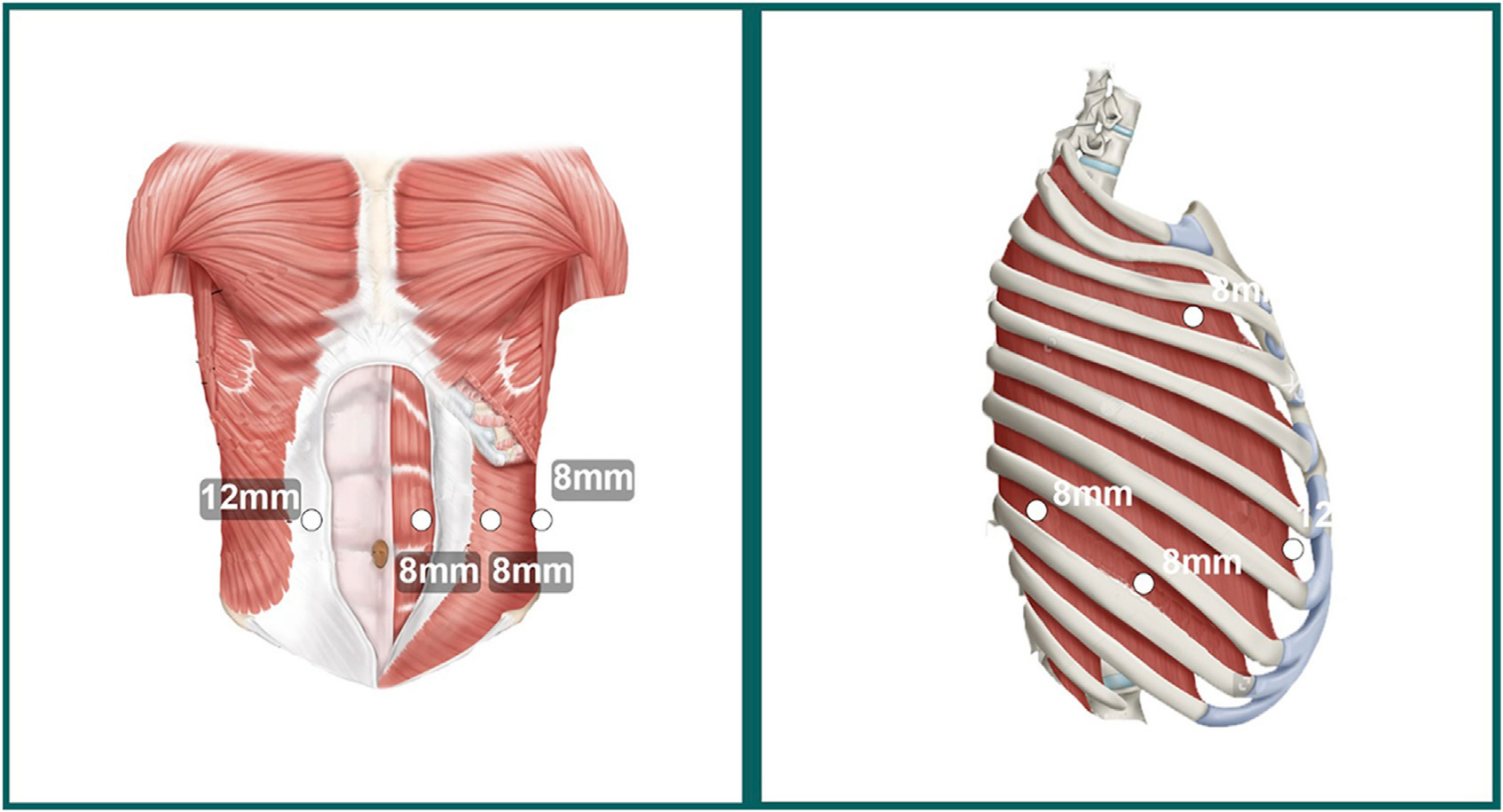
Robotic port placements for the abdominal and thoracic portions.

The abdominal cavity is inspected for metastatic disease, with the stomach elevated by using cigars to minimize tissue trauma and vasospasm while working around the gastroepiploic arteries. The stomach is retracted anteriorly and cephalad, exposing the greater omentum. Indocyanine green (ICG) is administered to confirm the location of right gastroepiploic artery. The vessel sealer is used to enter the lesser sac along the greater curvature, thus preserving the right gastroepiploic artery. A generous omental flap is created, and the short gastric arteries are divided. The dissection continues beyond the pylorus, thereby carefully freeing the posterior stomach and identifying the splenic artery and pancreas.

We perform a thorough abdominal (D2) and thoracic lymphadenectomy for oncologic staging purposes and to aid in identifying aberrant anatomy.

The duodenum is mobilized using a reverse Kocher maneuver. The gastrohepatic ligament is opened, and the lesser curvature of the stomach is mobilized with attention to any accessory left gastric artery. The esophageal hiatus is dissected by opening the phrenoesophageal ligament and skeletonizing the celiac axis. The left gastric artery is transected using white load robotic stapler (2.5 mm/45 mm). The esophagus is encircled using a Penrose drain for retraction, enabling dissection into the mediastinum. Robotic visualization aids high mediastinal dissection, which allows thorough lymphadenectomy through the abdominal approach extending past the inferior pulmonary vein. ICG confirms patency, and skin at each site is stapled for easy removal in Stage 2.

### Stage 2: Completion of the Robotic Ivor-Lewis Esophagectomy

#### Abdominal portion

The abdominal ports and liver retractor are again placed as discussed earlier. ICG is administered, and right gastroepiploic artery patency is confirmed. A window in the lesser omentum at the incisura, approximately 2 to 3 cm proximal to the pylorus, is created. A marking pen is used to measure a 2.5-cm conduit size. The measurement is marked along the greater curvature, and the stomach is retracted while the stapler is introduced through arm 1, with retraction by arms 3 and 4. The conduit is then created using green load staplers (4.3 mm/45 mm), with black load (4.6 mm/60 mm) reserved for more edematous tissue. Once the conduit transected, perfusion is confirmed using ICG. A marking pen is used to mark the conduit vertically to prevent twisting during delivery to the thorax. The gastric conduit is sewn to the gastroesophageal specimen with a 0 V-Loc suture (Medtronic), including a 19F Blake drain placed in the abdomen. When the specimen is delivered into the chest, the drain is positioned at the level of the anastomosis to reduce tension on the conduit suture.

#### Thoracic portion

The patient is positioned in the left lateral decubitus position, tilted anteriorly for optimal exposure of the posterior mediastinum, and single lung ventilation is initiated. Robotic port placements include a 12-mm port in the posterior eighth intercostal space for arm 1, an 8-mm port in the ninth intercostal space for arm 2, an 8-mm port in the third intercostal space for arm 3, and a 12-mm port in the inframammary region for arm 4, along with a 12-mm laparoscopic assist port at the diaphragm. After docking the robot, the inferior pulmonary ligament is incised to expose the pericardium, and lymph nodes are harvested as they are encountered. The pleura posterior to the esophagus is divided, and the dissection is extended cephalad, excising the subcarinal lymph node packet and encircling the azygous vein for division. A high esophagogastric anastomosis is created 20 to 25 cm from the incisors, with the esophagus transected just distal to the thoracic inlet by using the blade of the vessel sealer. The gastric conduit is delivered into the chest and divided to the appropriate length for a modified Collard anastomosis while ensuring adequate perfusion with ICG. A gastrotomy is created 4 cm from the end of the conduit, and the esophagus is stapled to form the posterior anastomosis, followed by a 2-layer hand-sewn closure of the anterior portion. After inspecting the anastomosis with an EGD and performing a leak test, a 20F chest tube and a 19F Blake drain are placed, and the specimen is delivered through the assistant port. Intercostal nerve blocks are administered, and a nasogastric tube is not routinely placed.

## Comment

Recently, the robotic platform has been used for esophagectomy, and it offers numerous advantages such as 3-dimensional visualization, wristed instruments, and advanced stapling technology. Although no definitive survival advantage has been established so far, promising results have emerged from high-volume, experienced centers.^6^^,^^7^ In our experience, this standardized approach to robotic Ivor-Lewis esophagectomy, using both sewn and stapled anastomosis, proves to be a safe and effective option for patients with midesophageal to distal esophageal cancers.
